# Supplementary material for: Bridging cone-beam CT and MRI to stopping power ratio maps: a modality-agnostic Brownian bridge approach to support robust adaptive proton therapy
Source: ArXiv. 2026 Sep 28:arXiv:2609.36339v1. Preprint. [Version 1] (PMC13648775)
Supplement: Supplementary file 1 [file NIHPP2609.36339v1-supplement-1.pdf]

Supplementary Materials

Supplementary materials for Brownian bridge methodology and additional results for model evaluation in HU

S1. Detailed expression of discretized reverse process of Brownian bridge

For discrete time step with a total number of time steps T, note $\mathbf{X}(t=0)=\mathbf{X}_0, \mathbf{X}(t=\mathrm{T})=\mathbf{X}_\mathrm{T}, \boldsymbol{\epsilon}\sim\mathcal{N}(0,\mathbf{I})$, $\boldsymbol{\epsilon_\theta}$ representing the Brownian bridge model that learns the difference between $\mathbf{X}_t$ and $\mathbf{X}_0$, then the reverse process can be discretized as:

$$\mathbf{X}_{t-1}=C_{xt}\mathbf{X}_\mathrm{t}-C_{yt}\mathbf{X}_\mathrm{T}-C_{\epsilon t}\boldsymbol{\epsilon_\theta}(\mathbf{X}_t,t)-C_{zt}\boldsymbol{z},\quad t=T,\ldots,2$$

where $\boldsymbol{z}\sim\mathcal{N}(0,\mathbf{I})$, and

$$C_{xt}=\frac{1}{C}(1+\frac{1}{\mathrm{T}\ln\frac{\mathrm{T}}{\mathrm{T}-t+1}})$$

$$C_{yt}=\frac{1}{C}(\frac{1}{\mathrm{T}-t+1}-\frac{t-1}{\mathrm{T}\,(\mathrm{T}-t+1)\ln\frac{\mathrm{T}}{\mathrm{T}-t+1}})$$

$$C_{\epsilon t}=\frac{1}{C}\frac{1}{\mathrm{T}\ln\frac{\mathrm{T}}{\mathrm{T}-t+1}}$$

$$C_{zt}=\frac{\lambda}{C}\sqrt{\frac{\mathrm{T}-t+1}{\mathrm{T}}}\sqrt{\frac{1}{\mathrm{T}}}$$

$$C=1-\frac{1}{\mathrm{T}-t+1}+\frac{1}{(\mathrm{T}-t+1)\ln\frac{\mathrm{T}}{\mathrm{T}-t+1}})$$

For t=1, $\mathbf{X}_0=\mathbf{X}_1-\boldsymbol{\epsilon_\theta}(\mathbf{X}_1,1)$.

Table S1. Summary of CT number-based quantitative metrics on synthetic SPR from CBCT and MRI using different models with statistical test results. Synthetic SPR maps were mapped back to HU numbers using the established Hounsfield look-up tables, then evaluated against ground truth CT in HU number. Metrics are reported in mean ± standard deviation [minimum, maximum]. P-values were adjusted using Bonferroni correction.

	Metric	Region	Modality-agnostic rBBrg	Uni-modality rBBrg	ResNet	Kruskal-Wallis P-value
**CBCT-based synthetic SPR results in HU (n=37)**	MAE (HU)	External	52 ± 6 [44, 65]	52 ± 6 [43, 64]	55 ± 6 [45, 75]	<0.05
		Soft tissue	38 ± 4 [33, 49]	38 ± 4 [33, 48]	40 ± 4 [34, 54]	1.00
		Bone	118 ± 17 [93, 161]	118 ± 17 [93, 161]	133 ± 17 [103, 172]	<0.05
		Air	126 ± 30 [85, 234]	128 ± 30 [84, 239]	137 ± 31 [88, 251]	0.35
	PSNR (d.B.)	External	32.2 ± 1.1 [29.6, 34.1]	32.2 ± 1.1 [29.5, 34.0]	31.9 ± 1.1 [29.5, 34.0]	1.00
	SSIM (a.u.)	External	0.89 ± 0.02 [0.83, 0.93]	0.89 ± 0.03 [0.82, 0.93]	0.86 ± 0.03 [0.80, 0.92]	<0.05
**MRI-based synthetic SPR results in HU (n=21)**	**Metric**	**Region**	**Modality-agnostic rBBrg**	**Uni-modality rBBrg**	**ResNet**	**Kruskal-Wallis P-value**
	MAE (HU)	External	84 ± 8 [73, 102]	85 ± 8 [74, 105]	85 ± 10 [72, 110]	1.00
		Soft tissue	48 ± 5 [39, 58]	49 ± 6 [39, 65]	45 ± 5 [36, 59]	0.43
		Bone	257 ± 27 [218, 327]	262 ± 27 [216, 316]	269 ± 34 [215, 347]	1.00
		Air	186 ± 39 [134, 277]	182 ± 40 [131, 272]	209 ± 52 [132, 357]	0.81
	PSNR (d.B.)	External	27.3 ± 1.0 [25.4, 28.8]	27.2 ± 0.9 [25.6, 28.7]	27.2 ± 1.1 [24.8, 28.7]	1.00
	SSIM (a.u.)	External	0.82 ± 0.02 [0.77, 0.85]	0.82 ± 0.02 [0.78, 0.85]	0.81 ± 0.03 [0.74, 0.86]	1.00

Table S2. Summary of post-hoc Dunn test results for metrics that showed significant Kruskal-Wallis test results comparing synthetic SPR (evaluated in HU) from CBCT and MRI across the three models.

**CBCT-based synthetic SPR results in HU (n=37)**	**Metric**	**Region**	**Modality-agnostic rBBrg** versus **Unimodality rBBrg**	**Modality-agnostic rBBrg** versus **ResNet**	**Uni-modality rBBrg** versus **ResNet**
	MAE (HU)	External	1.00	<0.05	<0.05
	MAE (HU)	Bone	1.00	<0.05	<0.05
	SSIM (a.u.)	External	1.00	<0.05	<0.05
